# Repression of intestinal transporters and FXR-FGF15 signaling explains bile acids dysregulation in experimental colitis-associated colon cancer

**DOI:** 10.18632/oncotarget.18885

**Published:** 2017-06-28

**Authors:** Lijuan Cao, Yuan Che, Tuo Meng, Shanshan Deng, Jun Zhang, Min Zhao, Wanfeng Xu, Dandan Wang, Zhichen Pu, Guangji Wang, Haiping Hao

**Affiliations:** ^1^ State Key Laboratory of Natural Medicines, Key Laboratory of Drug Metabolism & Pharmacokinetics, China Pharmaceutical University, Nanjing 210009, China

**Keywords:** bile acids, malabsorption, FXR, UFLC-Triple-TOF/MS, colitis-associated colon cancer

## Abstract

Bile acids (BAs) are important endogenous signaling molecules that play vital roles in the pathological development of various diseases including colitis-associated cancer (CAC). BAs were previously found dysregulated under conditions of CAC; however, the exact patterns and underlying molecular mechanisms remain largely elusive. Based on the development of a method for comprehensive analysis of BAs, this study aims to elucidate the dysregulation patterns and involved mechanisms in a typical CAC model induced by azoxymethane (AOM)/dextran sodium sulfate (DSS). CAC mice showed decreased BAs transformation in gut and glucuronidation in colon, leading to accumulation of primary BAs but reduction of secondary BAs in colon. CAC mice were characterized by an accumulation of BAs in various compartments except ileum, which is in line with repressed ileal FXR-FGF15 feedback signaling and the increased expression of hepatic CYP7A1. The compromised ileal FXR-FGF15 signaling was caused in part by the reduced absorption of FXR ligands including free and tauro-conjungated BAs due to the downregulation of various transporters of BAs in the ileum of CAC mice.

## INTRODUCTION

Bile acids (BAs) are a class of important endogenous bioactive substances that play pivotal roles in not only lipid digestion and absorption, but more importantly, serving as signaling molecules with systemic endocrine functions in a panel of important physiological and pathological processes [1–4]. Under physical conditions, primary BAs are synthesized in the liver and conjugated with glycine or taurine before secretion to intestinal tract where they are deconjugated and transformed to secondary BAs and reabsorbed in terminal ileum. Such an enterohepatic cycle leads to the production of more than 30 kinds of BA species. It has been shown that different BA species possess diverse bioactivities. Ursodeoxycholic acid (UDCA) has been widely used for treating ulcerative colitis (UC) complicated with primary sclerosing cholangitis [5, 6], preventing colitis conversion to colitis-associated cancer (CAC) [7]. In contrast, increased levels of deoxycholic acid (DCA) was reported to facilitate tumorigenesis of hepatocellular carcinoma [8] and CAC [9], despite that DCA has been repeatedly shown to be able to inhibit proinflammatory cytokines in monocytes/macrophages [10]. Thus, BA homeostasis should be tightly controlled to maintain their physiological functions. The dysregulation of BAs has been found closely associated with various diseases including CAC. It has been well known that patients with colon cancer are characterized with increased levels of BAs in feces [11, 12].

BA homeostasis is finely controlled by a complex machinery involving numerous nuclear receptors (NRs), enzymes, transporters, and the gut microbiota. BAs were initially identified as natural ligands of FXR [13–15], and more recently, several BA species including T-β-muricholic acid and UDCA were identified as FXR antagonists [16–18]. FXR, via regulating the expression of various enzymes and transporters involved in the synthesis, metabolism, and enterohepatic circulation of BAs, is thus a master regulator of BA homeostasis. Expression of BA synthetic enzymes, transporters and metabolic enzymes was found to be disturbed in *C. rodentium* infection-induced colitis mice [19]. Changes in the gut microbiota, which may lead to altered BA transformations in the gut and changes in circulating BAs, have also been implicated in inflammatory bowel disease [20]. BA malabsorption was considered the main mechanism of BA dysregulation in colon diseases [21, 22], whereas it is contrary to the fact that colitis patients are complicated with cholestasis [19, 23]. BA malabsorption in the conditions of colitis can be explained by the disturbed expression of various BA transporters in the ileum [22, 24]. More recently, we have verified that mice with DSS induced experimental colitis are characterized with both promoted metabolic excretion and increased de-novo hepatic synthesis of BAs, initiated by the activated intestinal PPARα-UGTs and compromised FXR-FGF15 signaling [25].

Chronic colitis characterized with unresolved inflammation is a high risk factor for the development of colon cancer. Some BA species like DCA and lithocholic acid (LCA) are believed to be cancer-promoting factors and thus BA dysregulation is widely considered to be closely associated with the development of colitis and lately CAC [26–28]. Moreover, FXR, the master BA regulator, was previously found involved in the pathogenesis of colon cancer [29]. All these evidence support an important role of FXR-BAs network in the pathologic development of CAC. However, it remains largely unknown about how BAs are dysregulated in the conditions of CAC. The present study was thus designed to determine the dysregulation patterns of BAs and the underlying mechanisms in CAC mice. A UFLC-Triple-TOF/MS method was established for the comprehensive analysis of various BA species in different compartments. Our results revealed that CAC mice were characterized with disturbed BA homeostasis as indicated by BA accumulation in most of the compartments and the upregulated expression of CYP7A1 in liver. We found that the elevated hepatic expression of CYP7A1 and thereafter the facilitated de-novo BAs synthesis might be explained by the decreased reabsorption of BAs due to the downregulation of various BA transporters, resulting in compromised FXR-FGF15 feedback signaling. Moreover, CAC mice showed decreased transformation in gut and glucuronidation in colon, leading to accumulation of primary BAs and reduced secondary BAs in colon.

## RESULTS

### Comprehensive profiling of BAs in CAC mice

**Figure 1:**
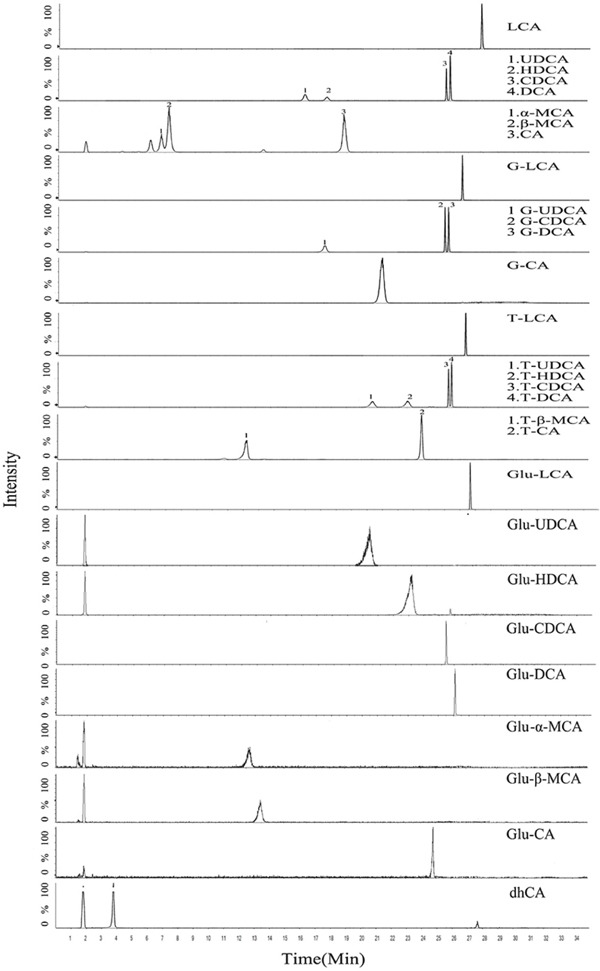
Representative chromatogram (XIC) of a mixure of BAs standards in serum matrix (free BAs, G-BAs, T-BAs, 250 ng/mL) including IS (50 ng/mL) and Glu-BAs products of *in vitro* incubation under the final chromatography and detection conditions .

In addition to the well-defined tauro- and glyco-conjugates, glucuronidation is another important kind of metabolism for BAs (compound structures were shown in Supplementary Figure 1). Although the overall rate of BA glucuronidation is low in comparison with tauro- and glyco-conjugation, it represents a major form in the urinal and fecal elimination of BAs since BA glucuronides do not undergo enterohepatic circulation [30]. Moreover, UDP-glucuronosyltransferases that catalyze BA glucuronidation are susceptible to regulation by various pathophysiological factors [31, 32]; therefore, it is important to monitor BA glucuronides in the pathological conditions for the intensive understanding of BA dysregulation patterns. To facilitate the process of simultaneous quantification of 28 BAs including the glucuronidation products, a method based on a UFLC-Triple-TOF/MS system was developed and validated. The chromatographic and mass spectrometric conditions were optimized to ensure good separation and sensitive detection of all the BA species (Figure 1). The correlation coefficient (r^2^) for all analytes spanning a wide range of concentration from 1 ng/mL to 2000 ng/mL was above 0.99. The method was fully validated for linearity, precision, accuracy and recovery, all of which fitted well with the requirements for the analysis of biosamples by FDA guidelines (Supplementary Tables 1-2).

As it is inconvinient to synthesize all the chemcial standards of BA glucuronides, we developed an approach for the relative quantification of BA glucuronides on the basis of using recombinant human UGTs incubating system. We hypothesized that the pure recombinant UGT catalyzed the formation of a sole BA glucuronide and thus the amount of glucuronide generated should be equal to that of the loss of BA precusors. A total of 12 kinds of human recombinant UGT enzymes (UGT1A1, 1A3, 1A4, 1A6, 1A7, 1A8, 1A9, 1A10, 2B4, 2B7, 2B15, and 2B17) were screened and UGT1A3 showed the strongest activity towards the glucuronidation of all the BAs. Thereafter, UGT1A3 was used for the following experiments. In each BA incubating system, only one metabolite was produced, which was consistent with the previous report [33]. Metabolite production is linear for at least 40 minutes in this incubation condition. Thus we assumed that the reduced amount of BA is equivalent to the generated amount of the metabolite. The putative ‘Glu-BA peak area-concentration’ equation was then deduced from the ‘BA concentration-incubation time’ curves and the ‘Glu-BA peak area–incubation time’ curves. The amount of various BA glucuronides in real biosamples was calculated from ‘Glu-BA peak area-concentration’ equation.

**Figure 2:**
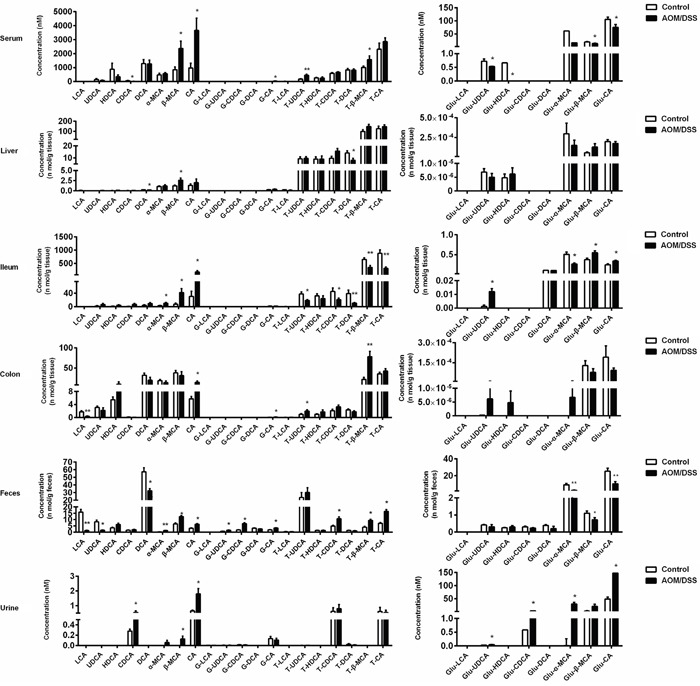
AOM/DSS-treated mice showed a significant disturbance of BA profile in serum, liver, ileum, colon, feces and urine compared with normal control mice, analyzed by UFLC-Triple/TOF-MS Results are expressed as mean ± S.E.M. of six mice. *P<0.05, **P<0.01 versus normal control, Student's *t*-test.

With this method, we were able to detect a panel of BAs, including unconjugated BAs in addition to those conjugated with glycine, taurine and glucuronic acid, in serum, ileum, liver, colon tissue, urine and feces of CAC mice. The successful development of a CAC model of mice induced by AOM/DSS was validated by hematoxylin and eosin staining (Supplementary Figure 2), which selectively developed in colon rather than in the ileum. The dysregulation patterns of BAs in CAC mice were comprehensively characterized (Figure 2). The serum levels of BAs, mainly primary BAs and tauro-conjugates, significantly increased in CAC mice in comparison with that of normal mice. Likewise, primary BAs and tauro-conjugates were all increased in the colon, urine and feces in CAC mice; however, most of the secondary BAs and glucuronides of BAs were generally decreased. Of particular interest, the level of all the tauro-conjugated BAs decreased in the ileum, implying an intricate dysregulation mechanism of BAs in CAC state.

### Gut microbiota-mediated BA transformation was decreased in CAC mice

**Figure 3:**
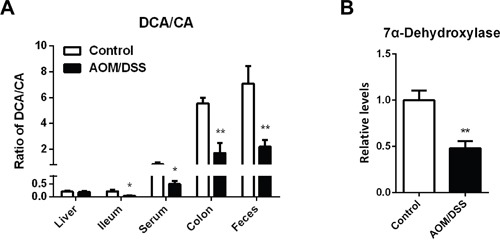
Specific accumulation of primary BAs in colon of CAC mice **(A)** Concentration ratio of DCA to CA in individual compartments, including serum, liver, ileum, colon and feces. **(B)** Relative DNA levels of 7α-dehydroxylase in cecum contents, normalized by weight of cecum contents. Data are represented as mean ± S.E.M. of six mice. *P<0.05, **P<0.01 versus normal control, Student's *t*-test.

Epidemiologic studies have suggested that intestinal flora disturbance has a major influence on CAC pathogenesis, and gut microbiota play vital roles in BA homeostasis. Intestinal anaerobic bacteria are responsible for 7α-dehydroxylation of primary BAs into formation of secondary BAs. It is evident that levels of primary BAs were elevated in gut compartments and excretes, while secondary BAs remarkably decreased (Figure 2). The transformation of primary BAs to secondary BAs, as indicated by ratio of DCA to CA, was significantly decreased (Figure 3A). *BaiCD* is a key functional gene in BAs 7α-dehydroxylation pathway in the genus *Clostridium*, which encodes stereospecific 3-dehydro-4-bile acid oxidoreductases recognizing 7α-hydroxy BAs [34]. In consist with decreased DCA/CA ratio, *baiCD* was markedly decreased in intestinal contents of CAC mice (Figure 3B).

### Increased de-novo synthesis of BAs in CAC mice

**Figure 4:**
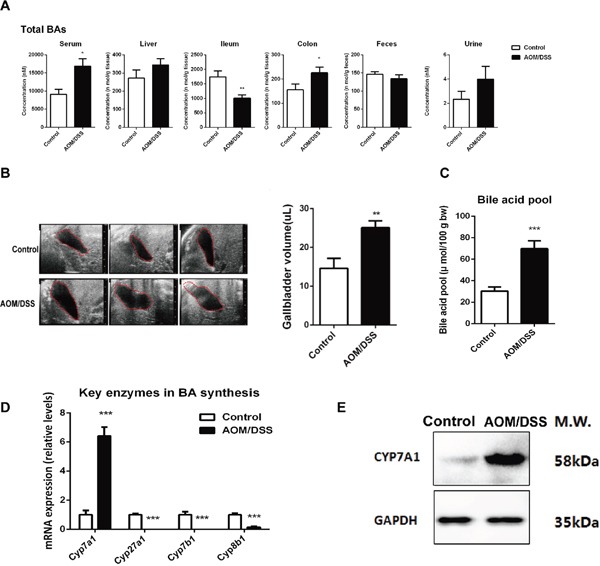
CAC mice complicated with excessive BA accumulation **(A)** Total BAs concentration was calculated by sum of individual BA concentrations in each department of serum, liver, ileum, coon, feces and urine of mice. **(B)** *In vivo* imaging of gallbladder by using abdominal ultrasonography, and quantitative results of gallbladder size from AOM/DSS mice compared with normal mice. **(C)** The BA pool size was analyzed by measuring total BAs in the whole enterohepatic system including liver, gallbladder, the entire small intestine and its contents, and the values were normalized by body weight. **(D)** The mRNA expression of *Cyp7a1*, *Cyp7b1*, *Cyp8b1* and *Cyp27a1* in liver. **(E)** Representative western blot analysis of CYP7A1 in liver from three independent experiments. Data are represented as mean ± S.E.M. of six mice. *P<0.05, **P<0.01, ***P<0.001 versus normal control, Student's *t*-test.

The levels of total BAs as estimated by summarizing all BA species were increased in all the compartments except the ileum (Figure 4A) and the total BAs concentrations in various compartments were validated by using a Mouse Total Bile Acids Kit (Crystal Chem, United States) (Supplementary Figure 3). Remarkable increased level of total BAs in BA pool analyzed by kit and the enlargement of gallbladder volume detected by *in vivo* abdominal ultrasonography(VisualSonics, Canada) suggests a promoted de-novo synthesis of BAs (Figure 4B, 4C). We thus determined the key enzymes involved in de-novo BAs synthesis. The mRNA and protein content of rate-limiting enzyme *Cyp7a1* in CAC mice were markedly upregulated (Figure 4D, 4E). In contrast, the mRNA levels of *Cyp27a1*, *Cyp8b1*, and *Cyp7b1* were markedly decreased.

### Repressed ileal FXR/FGF15 signaling in CAC mice

**Figure 5:**
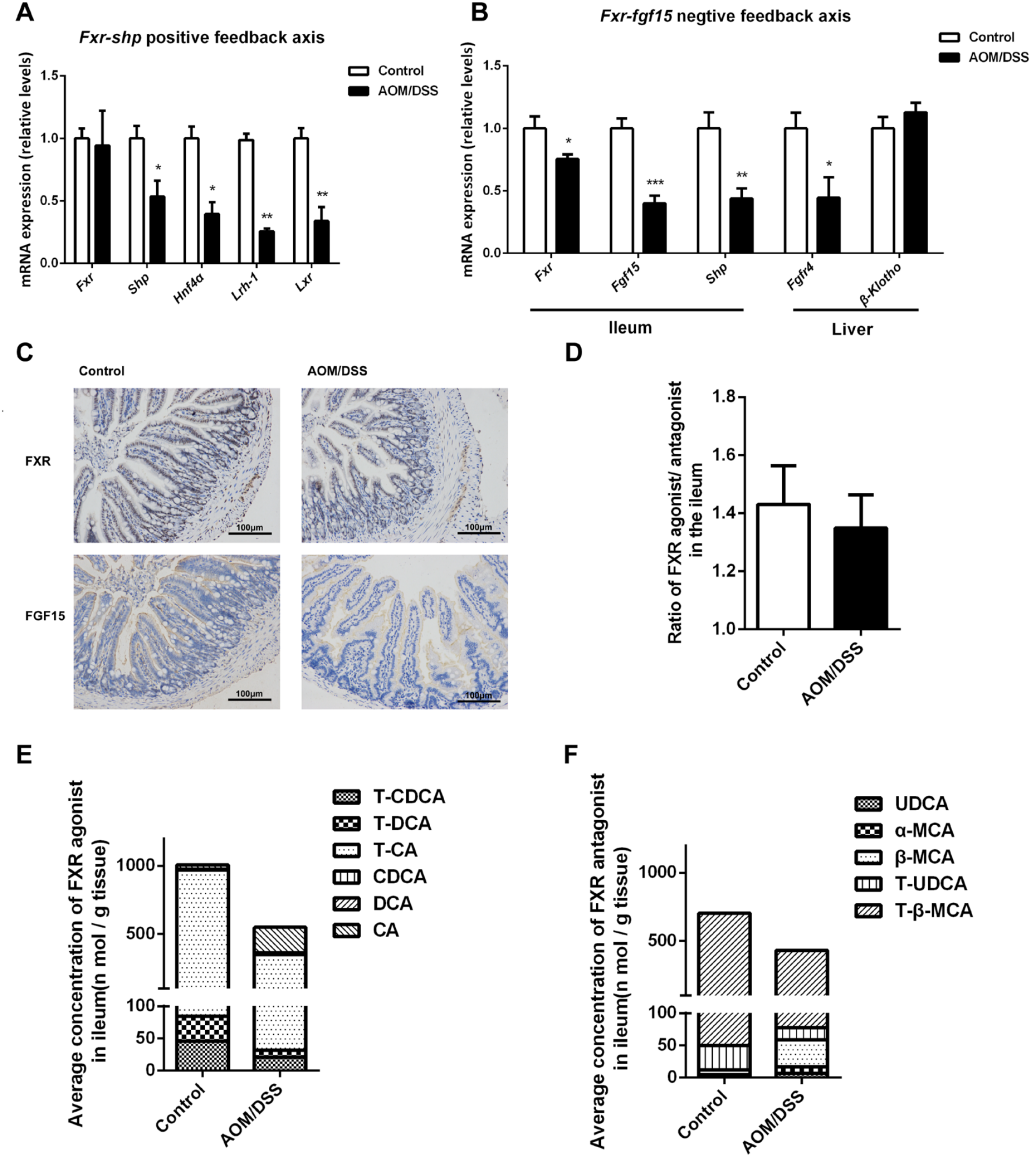
Repressed ileal FXR/FGF15 signaling leads to BAs over synthesis in CAC mice **(A)** ThemRNA expression of *Fxr*, *Shp*, *Hnf4α*, *Lrh-1* and *Lxr* in liver. **(B)** The mRNA expression of *Fxr*, *Fgf15*, *Shp* in the ileum, and *Fgfr4*, *β-Klotho* in liver. **(C)** Immunohistochemistry analysis of FXR and FGF15 in the terminal ileum of mice (scale bar, 100 μm). **(D)** The concentration ratio of summed FXR agonists/antagonists in the ileum of individual mouse. **(E)** Total concentration of FXR agonists in the ileum, calculated by sum of BA concentrations which can activate FXR. **(F)** Total concentration of FXR antagonists in the ileum, calculated by sum of BA concentrations which can activate FXR. Data are represented as mean ± S.E.M. of six mice. *P<0.05, **P<0.01, ***P<0.001 versus normal control, Student's *t*-test.

We next explored the underlying mechanisms involved in the upregulation of Cyp7a1 in CAC. Hepatic HNF4α and SHP were important transcription factors in controlling BA synthesis by trans-activating *Cyp7a1* promoter activity. We explored the Fxr/Shp positive feedback signaling in liver. Although *Fxr* mRNA expression was not affected, the mRNA levels of *Shp*, *Hnf4α, Lrh-1* and *Lxr* were all decreased in the liver of CAC mice (Figure 5A), suggesting that the upregulation of Cyp7a1 is unlikely due to the change of hepatic positive feedback signaling. Because ileal FXR-FGF15 signaling is an important determinant of hepatic CYP7A1 regulation, we thus turned to determine this ileal negative feedback signaling. As expected, ileum expression of *Fxr* and *Fgf15* was markedly decreased in CAC mice, demonstrated by both the mRNA and immunohistochemistry staining analysis. Moreover, the hepatic expression of FGF15 receptor *Fgfr4* was decreased, indicating compromised FXR-FGF15 feedback signaling in CAC mice (Figure 5B, 5C). As FXR is a ligand activated transcriptional factor and BAs are endogenous agonists of FXR for negative control of hepatic CYP7A1, we thus analyzed the intracellular levels of major BAs as either agonists (CDCA, CA, DCA and their tauro-conjugates) or antagonists of FXR (UDCA, α-MCA, β-MCA and their tauro-conjugates) in the ileum of CAC mice. The ileal levels of all these FXR agonists were decreased in CAC mice compared with normal control. Of interest, the antagonists levels in the ileum of CAC mice also decreased. These results suggest that FXR repression in the ileum of CAC mice was due to decreased concentration of its agonists (Figure 5D-5F).

### Dysregulated glucuronidation of BAs in CAC mice

**Figure 6:**
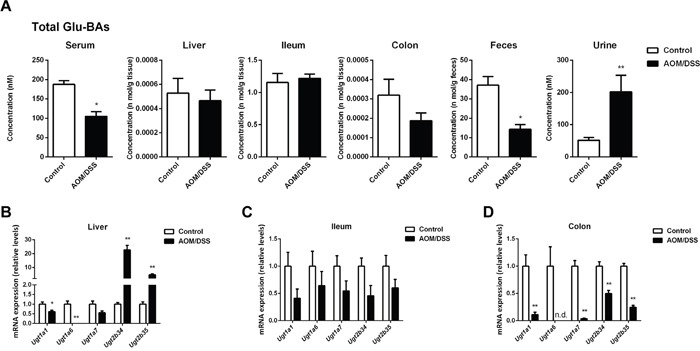
CAC mice showed dysregulated glucuronidation of BAs **(A)** Total concentration of glucuronide-conjugated BAs in individual compartments, including serum, liver, ileum, colon, feces and urine. **(B-D)** The mRNA expression of *Ugts* in liver, ileum and colon. Data are represented as mean ± S.E.M. of six mice. *P<0.05, **P<0.01 versus normal control, Student's *t*-test.

UGTs mediated glucuronidation plays an important role in the metabolic elimination of BAs and more recently, we have found that the facilitated intestinal BA glucuronidation was an initiating mechanism in compromising FXR-FGF15 signaling in DSS induced colitis. In contrast to the promoted glucuronidation of BAs in colitis, we found that in CAC mice the glucuronidation of BAs was largely repressed as indicated by the decreased levels of Glu-BAs in liver, colon and feces, as well as the mRNA expression of main *Ugts* (Figure 6).

### Disturbed BAs enterohepatic circulation in CAC mice

**Figure 7:**
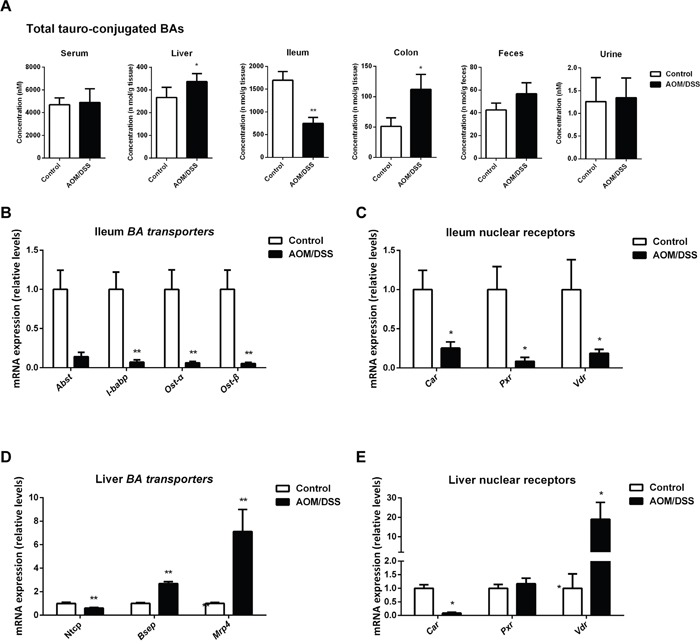
BA enterohepatic circulation is disturbed in CAC mice **(A)** Total concentration of tauro-conjugated BAs in individual compartments, including serum, liver, ileum, colon, feces and urine. **(B)** The mRNA expression of *Asbt*, *Ibabp*, *Ostα* and *Ostβ* in the ileum. **(C)** The mRNA expression of *Car*, *Pxr* and *Vdr* in the ileum. **(D)** The mRNA expression of *Ntcp*, *Bsep* and *Mrp4* in liver. **(E)** The mRNA expression of *Car*, *Pxr* and *Vdr* in liver. Data are represented as mean ± S.E.M. of six mice. *P<0.05, **P<0.01 versus normal control, Student's *t*-test.

Because UGTs mediated BAs glucuronidation in the ileum remained largely unchanged, we sought to determine the transporters involved in the uptake of BAs. We found taurine-conjugated BAs (T-BAs), which are dominant in the ileum, were markedly decreased in ileum while generally accumulated in other compartments of AOM/DSS-treated mice (Figure 7A). The uptake of T-BAs in the ileum depends on an active uptake system involving various transporters including the ileal apical sodium-dependent BA transporter (ASBT), the ileal BA-binding protein (IBABP), as well as the organic solute transporters α and β (OSTα and OSTβ). The mRNA levels of *Asbt*, *Ibabp*, *Ostα* and *Ostβ* were all decreased (Figure 7B). Consistently, the mRNA levels of various NRs involved in transcriptional regulation of these transporters showed a remarkable decrease (Figure 7C).

To further understand the enterohepatic circulation of BAs in CAC mice, we also checked the transporters in liver, including the major basolateral BA uptake transporter sodium taurocholate cotransporting polypeptide (NTCP) and canalicular efflux transporter bile salt export pump (BSEP), the multidrug resistance-associated protein 4 (MRP4). The mRNA level of *Ntcp* was significantly downregulated while *Bsep* and *Mrp4* were upregulated (Figure 7D), favoring a compensatory mechanism to eliminate over-synthesized BAs in liver of CAC mice. Main NRs including *Pxr*, *Vdr* and *Car* responsible for transcriptional regulation of these transporters were further explored (Figure 7E). *Car*, mainly responsible for transcriptional regulation of efflux transporters and *Ugt2b* family [35], is upregulated in liver of CAC mice, which is consistent with the upregulation of *Bsep*, *Mrp4* and *Ugt2b34/35*. In contrast, *Pxr*, mainly responsible for transcriptional regulation of uptake transporters and *Ugt1a* family [35, 36], was downregulated, leading to decreased *Ntcp* and *Ugt1a1/6*.

### Cyp27a1 upregulation explains dysregulation of BAs in Apc^Min/+^ mice

AOM/DSS induces a typical colitis associated colon cancer, representing a non-resolved inflammation driving development of cancer. Because not all the colon cancers in the clinic are associated with non-resolved inflammation, we hypothesized whether the BAs dysregulation pattern and underlying mechanisms determined from the AOM/DSS mice were or not applicable to the inflammation irrelative colon cancer. To this end, the profile of BAs and the associated signal pathways were determined in Apc^Min/+^ mice, a genetic intestinal tumorigenesis model [37], Apc^Min/+^ mice spontaneously develop intestinal tumors with aging (Supplementary Figure 6). In order to reach maximum instance rate, the mice were sacrificed 7 months after birth which showed tumor formation sporadically in the small intestine and colon as indicated by H&E staining (Supplementary Figure 4A). Apc^Min/+^ mice complicated BA accumulation to a minor extent than that in AOM/DSS mice, as evidenced from the data of BA pool size (Supplementary Figure 4B). Apc^Min/+^ mice were also characterized by the accumulation of BAs in the tumor tissues in both small intestine and colon (Supplementary Figure 4C, 4D). However, the underlying mechanism seems different from that of the AOM/DSS mice. In contrast to the significant decrease of BAs levels and related transporters mRNA expression in the ileum of AOM/DSS mice, no significant change was observed in the ileum of Apc^Min/+^ mice. The overall ratio of DCA and CA, representing the microbiota-mediated transformation from primary BAs to secondary BAs, exhibited no significant difference as well (Supplementary Figure 7). Moreover, minor upregulated level of hepatic *Cyp7a1* was observed than that in AOM/DSS mice, despite the drastic repression of ileal FXR/FGF15 signaling. Instead, Cyp27a1, the key enzyme involved in the alternative pathway of BAs synthesis, was found to be dramatically up-regulated in the liver of Apc^Min/+^ mice (Supplementary Figure 5).

## DISCUSSION

**Figure 8:**
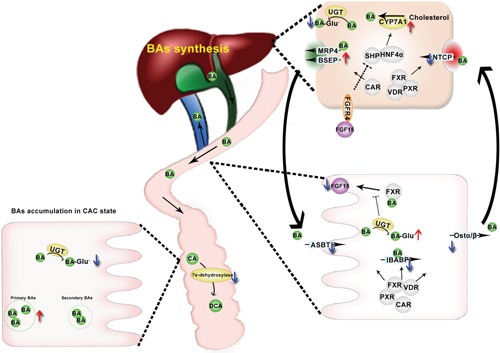
A summarize of BAs dysregulation in CAC mice CAC mice complicated with BA accumulation due to BA oversynthesis in liver and blocked enterohepatic circulation. In the ileum, NRs responsible for transcriptional regulation of BA transportation are found to be down regulated in the ileum of CAC mice, down regulation of BA transporters leads to BA malabsorption, combined with accelerated glucuronidation of BA, resulting in decreased FXR ligands concentration and FXR/FGF15 signaling in the ileum, promoting CYP7A1-mediated de-novo synthesis of BAs. BA malabsorption also leads to BA accumulation in colon, especially primary BAs, due to defected transformation of primary BAs to secondary BAs in CAC mice.

Although the accumulation of toxic BAs has long been recognized as a risk factor in CAC development, the exact dysregulation patterns and particularly the underlying mechanisms remain largely elusive. Based on developing a powerful method for the simultaneous quantification of major BAs and their glucuronides, this study contributes to a comprehensive understanding of BAs dysregulations and the mechanisms involved in CAC. CAC mice are characterized with the accumulation of BAs in most of the compartments except ileum, the compromised transformation of primary BAs to secondary BAs, the reduced ileal reabsorption, and the promoted hepatic de-novo synthesis. The repressive expression of ileal BAs uptake system including the NRs and transporters results in the decreased levels of FXR ligands and thereby the compromised FXR-FGF15 signaling (Figure 8).

More recently, we found that the promoted PPARα-UGTs axis is a driving factor in disturbing BAs homeostasis in DSS induced colitis mice. In contrast, we found herein in the AOM/DSS induced CAC mice the UGTs catalyzed glucuronidation of BAs was largely repressed as evidenced from both the quantification of BA glucuronides and the analysis of UGTs mRNA levels. The adaptive upregulation of UGTs in the early phase of inflammation may represent a protective mechanism in detoxifying BAs; however, it also leads to continuously promoted de-novo synthesis of BAs due to the upregulated hepatic CYP7A1. In the process of inflammation driving carcinogenesis, the expression of UGTs seems to be gradually repressed, which may contribute to the accumulation of toxic BAs particularly in the colon and thereafter promoting tumorigenesis. Our previous findings indicated that the intestinal transporters of BAs remained largely unchanged in colitis; in contrast, we found herein that the system involved in the ileal uptake of BAs, including various transporters and their upstream NRs, is markedly repressed. Therefore, the decreased retention levels of most BA species and particularly the taurine conjugates can be explained by the compromised active uptake system in CAC. These results strongly indicate that the enterohepatic circulation of BAs is decreased in CAC due to malabsorption. Moreover, because FXR is a ligand dependent transcriptional factor, its activation depends on the intracellular levels of various FXR ligands including agonists and antagonists, and FXR-feedback signaling repression in CAC mice was due to decreased BA agonists in the ileum. Therefore, the decreased uptake of BAs into the ileal epithelium may result in the compromised FXR-FGF15 signaling, which together with the downregulated hepatic *Fgfr4* leads to the upregulation of hepatic CYP7A1. Of interest, a panel of studies suggested that the ileal uptake system of BAs is also repressed in human patients with inflammatory bowel disease [38], contributing to the malabsorption of BAs [22, 39, 40]. Together, these studies indicate that the malabsorption of BAs due to the decreased expression of ileal transporters begins from the inflammatory state and may persist through the process of tumorigenesis, resulting in a prolonged hepatic over-synthesis of BAs and overload in the colon. BAs malabsorption in terminal ileum may further increase the quantity of BAs that escape the enterohepatic circulation and enter the colon. The exact mechanism underlying the downregulation of *Asbt* and other ileal BA uptake transporters and thereby BA malabsorption remains largely elusive. Inflammation might represent one of the important factors in this process since inflammatory cytokines could reduce the expression of *Asbt* and the expression of *Asbt* was shown downregulated in the patients of IBD [24, 41]. However, no significant inflammation was observed from the H&E staining of ileum in AOM/DSS mice in the current study. Since the regulatory network of *Asbt* expression *per se* is complex and remains largely unclear, it is hard for us to propose an exact mechanism of its downregulation in AOM/DSS mice. The levels of BAs, gut microbiota, and the transcriptional activity of its upstream receptors which were also found downregulated in AOM/DSS mice, may act together to regulate the expression of *Asbt*.

It was previously reported that the patients with BA malabsorption are usually accompanied with diarrhea and increased fecal excretion of BAs. Theoretically, the increased hepatic synthesis and decreased ileal reabsorption would like to result in increased fecal excretion of BAs. In our study, the concentration of BAs in the feces of AOM/DSS mice remains largely unchanged in comparison with that in the normal mice. However, it is important to note that the total amount of fecal excretion of BAs in AOM/DSS mice might be higher than that of the normal mice, because AOM/DSS mice are characterized with drastic diarrhea. Notably, AOM/DSS mice are also characterized with apparent BA accumulation as evidenced from the enlarged gallbladder and increased plasma concentration of BAs (Figure 4A, 4B). Therefore, it seems that enterohepatic circulation of BA in both directions (ileal reabsorption and gallbladder emptying) is compromised in AOM/DSS mice, which lead to BA accumulation and dramatic retention of BAs in the damaged colon.

In addition to the increased de-novo synthesis and the malabsorption of BAs, it seems that the relative BA compositions are changed in AOM/DSS mice. Cyp8b1, the key enzyme in controlling CA synthesis, was significantly decreased, which is supported by the decreased Lrh-1 level. However, the reduction of Cyp8b1 did not lead to significant decrease of CA/TCA. Thus, we extended to determine the transformation of CA by the gut microbiota. As expected, decreased transformation of CA to DCA in line with the decreased expression of *BaiCD*, a key functional gene in BAs 7α-dehydroxylation pathway in the genus *Clostridium* was observed. Along with the decreased DCA, LCA was also found decreased, supporting that the transformation of primary to secondary BAs is compromised in AOM/DSS mcie. Because the secondary BAs show anti-inflammatory activities while primary BAs are characterized with pro-inflammatory activities [42, 43], the increased primary to secondary BAs ratio may represent an important factor in facilitating the non-resolving inflammation in the pathological development of IBD and finally CAC.

Our further study indicated that BAs are also dysregulated in Apc^Min/+^ mice, as evidenced from the increased BA pool size and the drastic accumulation of BAs in tumor tissues. However, it is important to note that distinct dysregulation patterns and underlying mechanisms were observed between the Apc^Min/+^ mice and AOM/DSS mice. In contrast to the significant reduction of BAs in the ileum in AOM/DSS mice, the levels of BAs in the ileum of Apc^Min/+^ mice are significantly increased. This may be explained by that the tumor is developed in the ileum of Apc^Min/+^ mice but not AOM/DSS mice. The ileal FXR/FGF15 signaling is also repressed, which is likely due to the decreased expression and transcriptional activity of FXR itself. Of interest, the repressed FXR/FGF15 signaling dose not lead to significant increase of hepatic *Cyp7a1* expression. Instead, *Cyp27a1*, a key enzyme in the alternative synthetic pathway of BAs, is significantly upregulated. Consistently, it was previously reported that *Cyp27a1* is highly expressed in the patients with colorectal cancer, hinting to a causal link among *Cyp27a1*, BAs, and the development of colon cancer [44]. Taking together, our study indicates that although the dysregulation patterns and mechanisms of BAs may vary between the inflammation relative and irrelative colon cancer, the accumulation of BAs and particularly in the tissues where tumor develops is a common phenotype, supporting an important role of BAs dysregulation in the pathological development of colon cancer.

## MATERIALS AND METHODS

### Chemicals and reagents

HPLC-grade methanol and acetonitrile were purchased from Fisher Scientific (Nepean, Ont., Canada). Ultrapure water was prepared by the Milli-Q Ultrapure water purification system (Millipore, Bedford, MA, USA). AOM was obtained from Sigma-Aldrich (St. Louis, MO, USA), and DSS (MW 36000-50000) was purchased from MP Biomedicals (Santa Ana, CA). Ammonium acetate, formic acid, glacial acetic acid and the other reagents (analytical grade) were purchased from Nanjing Chemical Factory (Nanjing, China). ‘Mouse Total Bile Acids Kit’ was purchased from Crystal Chem Inc (Downers Grove, United States). ‘MiniBEST Bacterial Genomic DNA Extraction Kit’ was from TaKaRa Bio Inc (Dalian, China). Anti-FXR, FGF15 and CYP7A1 antibodies were all purchased from Santa Cruz Biotechnology (Santa Cruz, CA, USA), and anti-GAPDH antibody was from Bioworld Technology (St. Louis Park, MN, USA).

CA, CDCA, DCA, and LCA, as well as their glycine [38] and taurine [45] conjugates, G-CA, G-CDCA, T-β-MCA, T-CA, T-CDCA, T-DCA, and internal standard dehydrocholic acid (dhCA) were obtained from Sigma-Aldrich (St. Louis, MO, USA). α-MCA, UDCA, HDCA, G-LCA, G-UDCA, G-DCA, T-β-MCA, T-UDCA, T-HDCA and T-LCA were purchased from Steraloids, Inc. (Newport, Rhode Island, USA).

### Animals and treatments

Specific pathogen free male C57BL/6 mice were obtained from Shanghai SLAC Laboratory Animal Co., Ltd (Shanghai, China) at the age of 5 weeks, and maintained at Animal Facility according to protocols approved by the Review Committee of Animal Care and Use of China pharmaceutical university. On arrival, all mice were randomized and transferred to plastic cages, and were given free access to drinking water and diet, under controlled conditions of humidity (50±10%), light (12/12 hours light/dark cycle), and temperature (25±2°C). All mice were quarantined for one week before starting the experiment.

An experimental mouse model of CAC was established following the previous protocol [46]. In detail, colon carcinogenesis was induced by intraperitoneally injection with 12 mg/kg of AOM dissolved in physiological saline. Five days later, mice were treated with three administration cycles of DSS. Each cycle was composed of 7 days' consumption of drinking water containing 2% DSS followed by 14 days' consumption of tap water. To obtain urine and feces samples at the last day, mice (N=6) were placed in metabolic cages and samples were collected for 24 hours. The animals were sacrificed at the 10^th^ week; serum and tissue samples were harvested freshly (tissues were washed with phosphate buffer solution to minimize the contribution of blood and intestinal contents to the measured tissue profile), frozen in liquid nitrogen, and stored at −80°C until analysis.

Male Apc^Min/+^ mice and C57BL/6 background wide-type (WT) mice were purchased from Nanjing BioMedical Research Institute of Nanjing University (NBRI). All mice were maintained in pathogen-free facilities until being euthanized at 7 month-old for determining metabolic dysregulation mechanisms of BA.

All animal studies were conducted according to protocols approved by the Review Committee of Animal Care and Use of China Pharmaceutical University and have been carried out in accordance with the Declaration of Helsinki.

### Histopathological analysis and immunohischemical staining

The entire colon was excised and opened longitudinally, flushed with PBS and fixed in 10% formaldehyde, then embedded in paraffin and 5 μm-thick sections were stained with hematoxylin and eosin (H&E), and then evaluated by an experienced investigator in a double-blinded manner with an optical microscopy. Ileum tissues were preserved in 10% formalin and embedded in paraffin; tissue sections were then immunolabelled with anti-FXR antibody and anti-FGF15 antibody individually overnight at 4°C and subsequently incubated with HRP-conjugated second antibody. Each slice was imaged by Leica DMI 3000B inverted fluorescence microscope (Leica Microsystems, Bensheim, Germany) and the protein content was analyzed with Image pro-plus 6.0 software.

### Quantitative reverse transcription PCR

Total tissue RNA extraction was performed using the RNAiso Plus reagent (TaKaRa Biotechnology Co., Ltd, Dalian, China) according to the manufacturer's protocol. cDNA was generated from 500 ng total RNA using Super Script II Reverse Transcriptase (Applied Biosystems, Carlsbad, California, USA). qRT-PCR analysis was carried out using SYBR green PCR master mix (Biorad) and analyzed on a real time PCR cycler (Applied Biosystems, Carlsbad, California, USA). Simultaneous quantification of *Gapdh* mRNA was used as internal control. Sequences for primers are listed in Supplementary Table 3.

Genomic DNA from the whole cecum contents was extracted from cecum contents by using a Bacterial Genomic DNA Extraction Kit; qRT-PCR analysis was carried out for determination of bacterial gene *BaiCD* (forward 5′-CAGCCCRCAGATGTTCTTTG, reverse 5′-GCATGGAATTCWACTGCYTC), universal primer forward 331F (5′-TCCTACGGGAGGCAGCAGT) and reverse 979R (5′-GGACTACCAGGGTATCTATCCTGTT) were used for quantification of total bacterial gene as internal control. The expression levels were normalized by weight of total cecum contents.

### SDS-PAGE and western blotting

Tissues were lysed in RIPA lysis buffer (Beyotime, Shanghai, China), supplemented with 2 mM PMSF. Total protein (60 μg) was resolved by SDS-PAGE and transferred to a PVDF membrane. Membranes were blocked with 5% skim milk in TBS containing 0.1% Tween 20. The bands were reacted with primary antibodies diluted in blocking buffer overnight at 4°C. The membranes were washed and then incubated with secondary antibody (anti–rabbit horseradish peroxidase) diluted 1:10,000 in 5% BSA in TBS containing 0.1% Tween 20 for 1 h at room temperature. The membranes were washed and then chemiluminescense was detected with an image analyzer (LAS-1000; FujiFilm). Antibody for GAPDH and second antibody were obtained from Bioworld Technology (Nanjing, China).

### LC-MS analysis of BAs

The chromatographic system consisted of a Shimadzu (Kyoto, Japan) UFLC system consisting of an LC-30AD binary pump, a DGU-20A5 degasser, a SIL-30AC autosampler and a CTO-20A column oven. The chromatographic separation was carried out on a ZOEBAX Eclipse Plus C_18_ column (2.1 × 150 mm, 3.5 μm) (Agilent Technologies, USA), protected by an Security Guard (Phenomenex Inc. CA. USA). The mobile phase (delivered at 0.2 mL/min) consisted of (A) 2.6mmol/L ammonium acetate in water (adjusted to pH 6.8 with ammonium hydroxide) and (B) acetonitrile. The total running time was 34 min with a gradient elution: initial 20% B for 5 min, linear gradient 20–25% B from 5 to 10 min and retained until 20 min then linear gradient 25–55% B from 20 to 25 min and retained until 29 min, and then quickly returned to initial 20% B in 2 min and maintained for a further 3 min for column balance. The injection volume was 5 μL.

Mass spectra were obtained using an AB SCIEX Triple TOF™ 5600 mass spectrometer equipped with a QJet™ ion guide and accelerated by a LINAC^®^ collision cell (AB Sciex, Foster City, CA, USA) with an atmospheric pressure chemical ionization probe in a Turbo V™ ion source. The mass spectrometer was operated in the negative ion mode, scanning from m/z 300 to 800. The spray voltage was set at 5500 V, nebulizer gas (Gas 1), 50 psi; heater gas (Gas 2), 50 psi; curtain gas, 30 psi, and the capillary temperature was 400°C. The following optimized condition was used: declustering potential, 70 V; collision energy, 40eV. Nitrogen was used as nebulizer and auxiliary gas. Recalibration was carried out by Easy-Mass Accuracy^®^ device before analysis. Analyst 1.4.2 software (AB Sciex, Foster City, CA, USA) was used for system control, PeakView software for data exploring, and quantification data were acquired with MultiQuant software. The fragmentation process of BAs was described previously [25, 47, 48].

For feces samples, approximately 100 mg of feces was homogenized in 500 μL of 70% ethanol, then ultrasonic extracted for 4 hours at 55°C, and centrifuged at 4,000 g for 10 min. 100 μL of the extract solution was diluted with 500 μL of 0.1‰ formic acid-spiked with 1 μL IS, vortexed, and loaded onto Oasis-HLB cartridges which were pre-conditioned with 1 mL MeOH and followed by 1 mL H_2_O. Loaded cartridges were washed with 1 mL H_2_O and eluted with 1.5 mL MeOH. The eluate was evaporated under vacuum at 45°C and reconstituted in 100 μL methanol by vortexing. Tissue, urine and serum samples were also prepared by SPE method. Approximately 200 mg of tissue was ultrasonic extracted in 500 μL of 70% ethanol for 4 hours at 55°C, and the following preparation steps was in the same way with fecal samples. Urine/Serum samples (100 μL) were directly diluted with 500 μL of 0.1‰ formic acid-spiked with 1 μL IS and cleaned up the same as feces samples by SPE method. Extraction recoveries were determined for each quality control (QC) point in serum matrix as from the ratio of the analyte peak area in samples spiked before extraction compared to the corresponding peak area in untreated samples prepared in neat solution.

BAs-free mouse matrix was purified using activated charcoal to remove endogenous BAs. Such BAs-free matrix was used as biological matrix in this study. To be specific, 1mL of biofluid or extracted solution of tissue was mixed with 50 mg of activated charcoal and the mixture was shaken continuously on an orbital shaker for 4 hours at room temperature. After centrifugation at 25,000 g for 20 min, the supernatant of purified matrix was transferred to clean tubes and kept at −20°C until use. No significant matrix effect was observed for all the biosamples analyzed and thus serum matrix was used for the full methodology validation.

In the case of the lack of standard Glu-BAs, a relative quantification method was developed by the use of recombinant human UGT1A3 incubation system. In detail, a 0.2mL reaction mixture containing 0.1 mg/mL protein, 2 mM uridinediphosphoglucuronic acid, 5 mM MgCl_2_, 0.025 mg/mL alamethicin and 0.8 μg/mL BA in 50 mM Tris-HCl buffer solution (pH7.4) was incubated at 37°C for 0, 5, 10, 20, 30, 40, and 50 min, respectively. After incubation, the reaction was terminated by instantly freezing method. The incubation mixture was then pretreated by SPE method before quantification as described above.

### BA pool size analysis

Total BAs of mouse were extracted from the whole liver, gallbladder, intestine and its contents by homogenization and ultrasonic extraction at 55°C for 30min in 25mL of 70% (v/v) ethanol solution. The homogenate was centrifuged at 16,000g for 10 min. The supernatant was then analyzed by a Mouse Total Bile Acids Kit (Crystal Chem, Downers Grove, USA). The BA pool size per mouse was normalized by body weight.

### *In vivo* abdominal ultrasonography

Mice were anaesthetized with 2% isoflurane and transferred to the preparation station. Animal fur was shaved from the mice abdominal area. Gallbladder images was detected with Vevo2100(Ultrasonics, Canada) following the standard protocols.

### Statistical analysis

The data are representative of three independent experiments, and were statistically analyzed by GraphPad Prism 6 software (GraphPad), using the build-in Student *t*-test as indicated in the individual figure legends where statistical significance was defined as P value < 0.05. Data are shown as the mean ± SEM.

## SUPPLEMENTARY MATERIALS FIGURES AND TABLES





## Abbreviations

BAs: bile acids
LCA: lithocholic acid
UDCA: ursodeoxycholic acid
HDCA: hyodeoxycholic acid
CDCA: chenodeoxycholic acid
DCA: deoxycholic acid
CA: cholic acid
MCA: muricholic acid
G-BAs: glycine-conjugated bile acids
T-BAs: taurine-conjugated bile acids
Glu-BAs: glucuronidated bile acids
dhCA: dehydrocholic acid
Bsep: bile salt export pump
Asbt: apical sodium-dependent bile acid transporter
Mrp: multidrug resistance protein
Ntcp: Na+/taurocholate cotransporting polypeptide
Ostα/β: organic solute transporter alpha/beta
IBABP: ileal bile acid binding protein
NRs: nuclear receptors
FXR: farnesoid X receptor
FGF: fibroblast growth factor
SHP: small heterod imer partner
PXR: pregnane X receptor
VDR: vitamin D receptor
CAR: constitutive androstane receptor
AOM: azoxymethane
DSS: dextran sodium sulfate
CAC: colitis-associated colon cancer
UFLC: ultra-flow liquid chromatography
MDF: mass defect filter
CYP: cytochrome P450
UGT: UDP-glucuronosyltransferase.
