# Supplementary material for: Repression of intestinal transporters and FXR-FGF15 signaling explains bile acids dysregulation in experimental colitis-associated colon cancer
Source: Oncotarget. 2017 Jun 28;8(38):63665–79. doi: 10.18632/oncotarget.18885 (PMC5609951; doi:10.18632/oncotarget.18885)
Supplement: Supplementary file 1 [file oncotarget-08-63665-s001.pdf]

Repression of intestinal transporters and FXR-FGF15 signaling explains bile acids dysregulation in experimental colitis-associated colon cancer

SUPPLEMENTARY MATERIALS

Bile acids	Abbreviations	R1	R2	R3	R4	R5
Cholic acid	CA	OH	H	H	H	OH
α-muricholic acid	α-MCA	H	OH	H	H	OH
β-muricholic acid	β-MCA	H	OH	H	OH	H
Ursodeoxycholic acid	UDCA	H	H	H	OH	H
Hyodeoxycholic acid	HDCA	H	H	OH	H	H
Chenodeoxycholic acid	CDCA	H	H	H	H	OH
Deoxycholic acid	DCA	OH	H	H	H	H
Lithocholic acid	LCA	H	H	H	H	H

Bile acids	R6
Unconjugated	COOH
Glycine-conjugated	$CONHCH_2COOH$
Taurine-conjugated	$CONH(CH_2)_2SO_3H$
Glucuronyl-conjugated	$COOC_6H_9O_6$

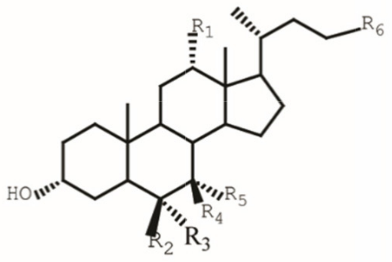

Supplementary Figure 1: Backbone and side chain structures of the 8 major BAs, as well as their glycine, taurine and glucuronic acid conjugates in mice.

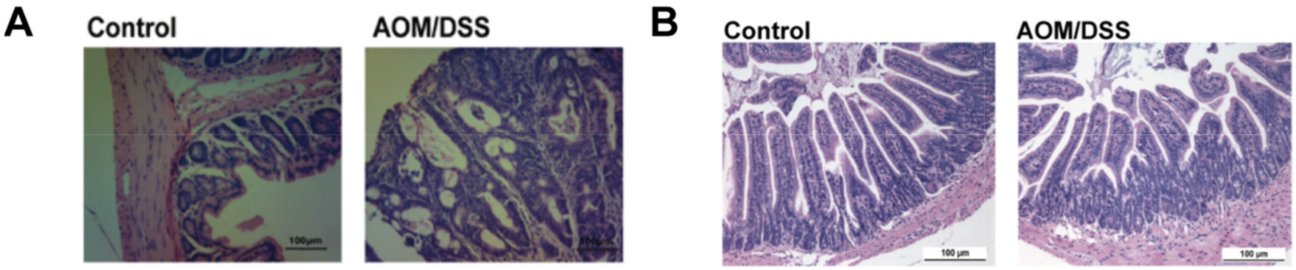

Supplementary Figure 2: AOM/DSS induced CAC mouse model was validated by hematoxylin and eosin staining of colon and ileum tissues (Scare bar, 100µm). **(A)** Hematoxylin and eosin staining of colon tissues from AOM/DSS induced CAC mouse model. **(B)** Hematoxylin and eosin staining of ileum tissues from AOM/DSS induced CAC mouse model.

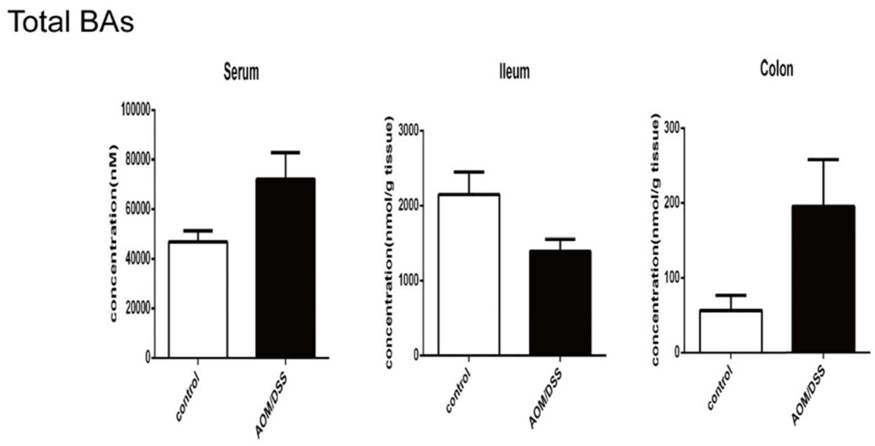

Supplementary Figure 3: Total BAs concentration was detected with Mouse Total Bile Acids Kit in serum, ileum and colon of CAC mice.

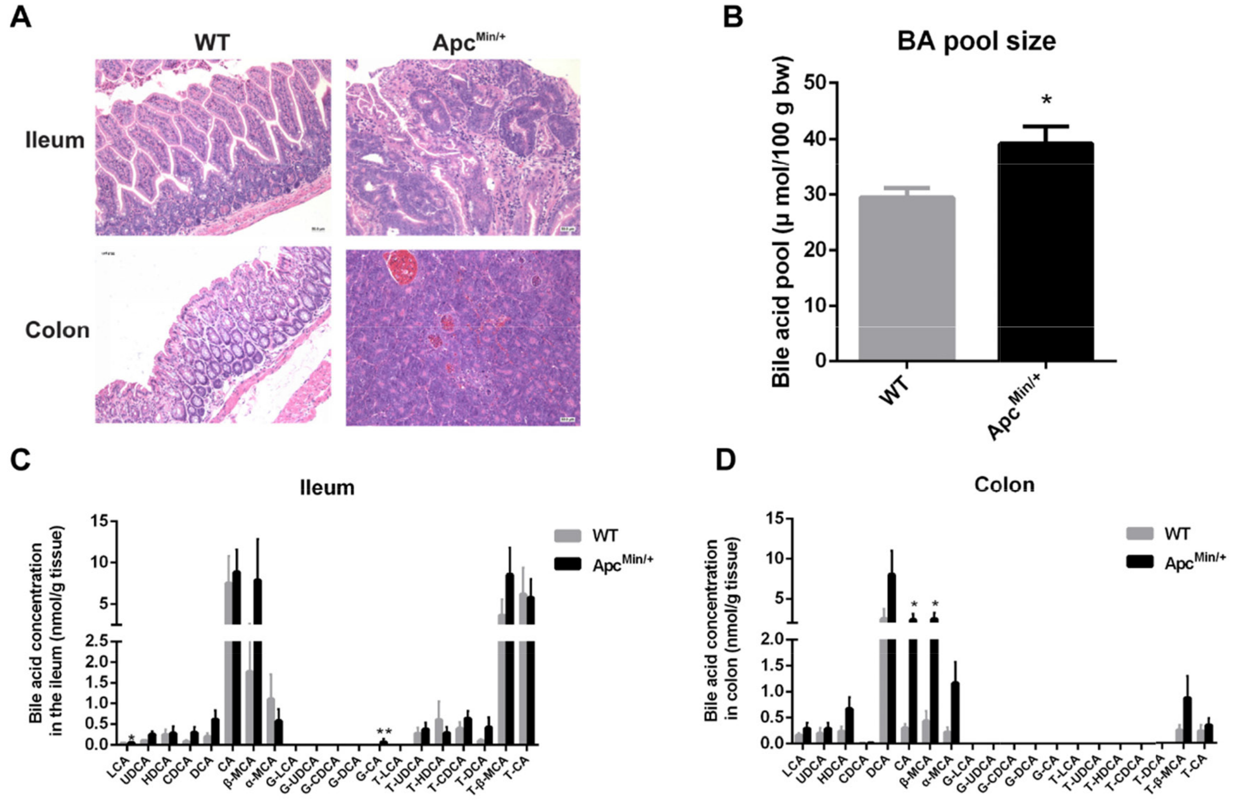

Supplementary Figure 4: Excessive BA accumulation in Apc$^{Min/+}$ mice. (A) Tumor formation in intestine and colon of Apc$^{Min/+}$ mice was validated by H&E staining (Scare bar, 50μm). **(B)** The BA pool size was analyzed by measuring total bile acids in the whole enterohepatic system including liver, gallbladder, the entire small intestine and its contents, and the values were normalized by body weight. **(C-D)** Apc$^{Min/+}$ mice showed a significant accumulation of BA in ileum and colon compared to WT mice, analyzed by UFLC-Triple/TOF-MS. Results are expressed as mean ± S.E.M. of six mice. *P<0.05, **P<0.01 versus WT control, Student's *t*-test.

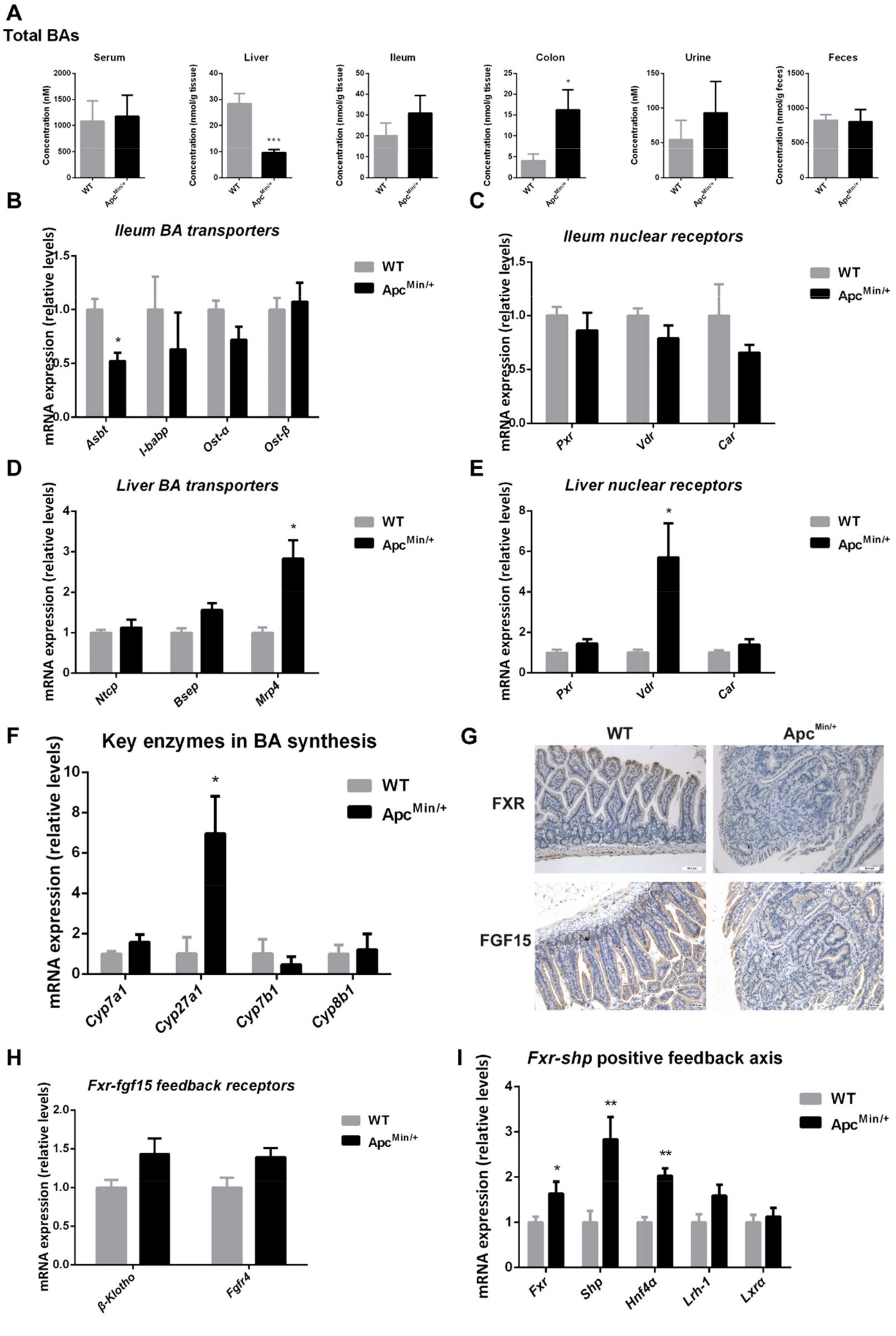

Supplementary Figure 5: CYP27A1 upregulation leads to BA accumulation in $Apc^{Min/+}$ mice. **(A)** Total BAs concentration was calculated by sum of individual BA concentrations in each department of serum, liver, ileum, coon, feces and urine of mice. **(B)** The mRNA expression of *Asbt*, *Ibabp*, *Ostα* and *Ostβ* in the ileum. **(C)** The mRNA expression of *Car*, *Pxr* and *Vdr* in the ileum. **(D)** The mRNA expression of *Ntcp*, *Bsep* and *Mrp4* in liver. **(E)** The mRNA expression of *Car*, *Pxr* and *Vdr* in liver. **(F)** The mRNA expression of *Cyp7a1*, *Cyp7b1*, *Cyp8b1* and *Cyp27a1* in liver. **(G)** Immunohistochemstry analysis of FXR and FGF15 in the terminal ileum of mice (scale bar, 50 μm). **(H)** The mRNA expression of *β-Klotho* and *Fgfr4* in liver. **(I)** The mRNA expression of *Fxr*, *Shp*, *Hnf4α*, *Lrh-1* and *Lxrα* in liver. Data are represented as mean ± S.E.M. of six mice. $^*P<0.05$, $^{**}P<0.01$ versus WT control, Student's *t*-test.

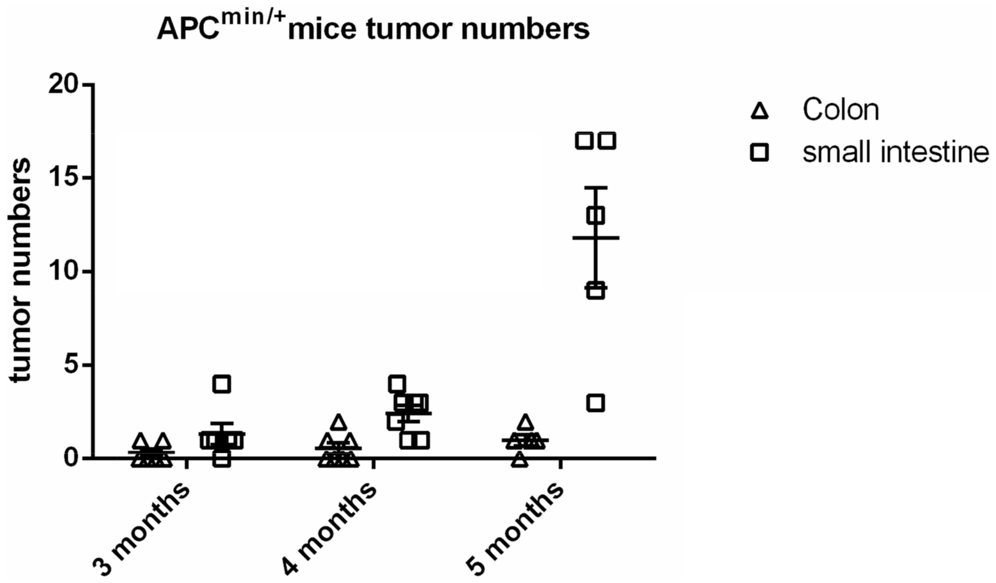

Supplementary Figure 6: Tumor numbers in colon and small intestine in APC$^{min/+}$ mice.

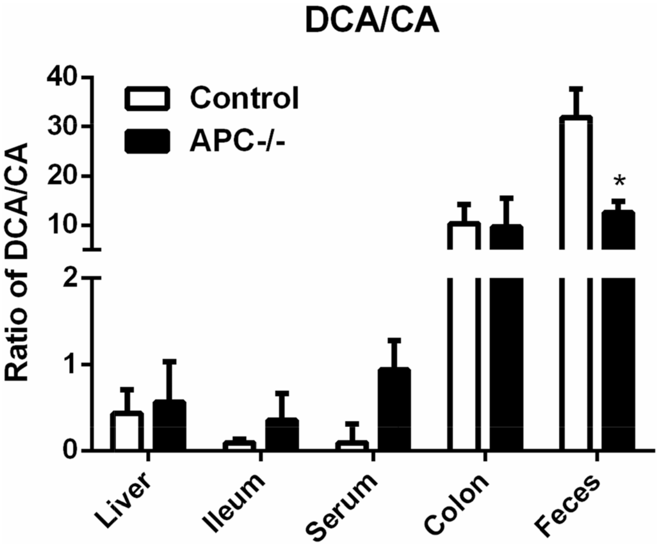

Supplementary Figure 7: Concentration ratio of DCA to CA in individual compartments, including liver, ileum, serum, colon and feces in APC$^{Min/+}$ mice.

Supplementary Table 1: Calibration curves (y = bx + a, r^2), detection and quantification limits for the available BAs determined by UFLC-Triple-TOF/MS (n =6)

Bile acids	Dynamic range (0.001-2μg/mL) y = bx + a, r^2		LOD (ng/mL)	LOQ (ng/mL)
	0.001-0.1μg/mL (R^2 0.997~0.999)	0.1-2μg/mL (R^2 0.990~0.999)		
LCA	y = 24.08x + 0.034;	y = 18.53x + 0.626;	0.1	1
UDCA	y = 10.02x + 0.005;	y = 8.203x + 0.011;	0.1	1
HDCA	y = 8.044x + 0.002;	y = 6.353x + 0.048;	0.1	1
CDCA	y = 12.54x + 0.008;	y = 10.27x - 0.013;	0.1	1
DCA	y = 16.95x + 0.011;	y = 13.08x + 0.133;	0.1	1
CA	y = 9.018x + 0.003;	y = 7.114x + 0.101;	0.1	1
α-MCA	y = 6.605x + 0.001;	y = 7.874x + 0.191;	0.1	1
β-MCA	y = 8.970x + 0.002;	y = 13.66x + 0.324;	0.1	1
G-LCA	y = 30.02x + 0.112;	y = 24.63x - 0.214;	0.1	1
G-UDCA	y = 7.907x + 0.005;	y = 7.979x - 0.363;	0.1	1
G-DCA	y = 15.77x + 0.032;	y = 16.20x - 0.523;	0.1	1
G-CA	y = 7.391x + 0.008;	y = 7.178x - 0.282;	0.1	1
T-LCA	y = 21.26x + 0.070;	y = 14.71x + 0.574;	0.1	1
T-UDCA	y = 7.286x + 0.007;	y = 7.480x - 0.390;	0.1	1
T-HDCA	y = 8.081x + 0.012;	y = 8.216x - 0.334;	0.1	1
T-CDCA	y = 9.555x + 0.018;	y = 9.710x - 0.403;	0.1	1
T-DCA	y = 12.00x + 0.015;	y = 12.96x - 0.379;	0.1	1
T-CA	y = 6.121x + 0.009;	y = 5.977x - 0.198;	0.5	1
T-β-MCA	y = 6.145x + 0.008;	y = 5.885x - 0.211;	0.5	1

Supplementary Table 2: Precision, accuracy and SPE recovery for quantification of available BAs

See Supplementary File 1

Supplementary Table 3: Primer sequences for qRT-PCR (mice)

See Supplementary File 1
