# Supplementary material for: Repression of intestinal transporters and FXR-FGF15 signaling explains bile acids dysregulation in experimental colitis-associated colon cancer
Source: Oncotarget. 2017 Jun 28;8(38):63665–79. doi: 10.18632/oncotarget.18885 (PMC5609951; doi:10.18632/oncotarget.18885)
Supplement: Supplementary file 2 [file oncotarget-08-63665-s002.docx]

**Supplementary Table 2:** **Precision, accuracy and SPE recovery for quantiﬁcation of available BAs.**

| **Bile acids** | **Norminal concentration**  **(ng/mL)** | **Intra-day (n=6)** | | **Inter-day (n=6)** | | **SPE recovery**  **(%)** |
| --- | --- | --- | --- | --- | --- | --- |
|  |  | **Accueracy**  **(%)** | **CV**  **(%)** | **Accueracy**  **(%)** | **CV**  **(%)** |  |
| LCA | 5 | 106.53 | 4.62 | 97.66 | 4.66 | 89.13 |
|  | 100 | 99.99 | 4.10 | 99.30 | 2.88 | 90.13 |
|  | 2000 | 99.68 | 2.98 | 100.09 | 3.84 | 87.77 |
| UDCA | 5 | 113.54 | 7.63 | 112.09 | 2.48 | 97.01 |
|  | 100 | 100.13 | 1.21 | 100.10 | 3.12 | 97.44 |
|  | 2000 | 100.74 | 4.31 | 101.22 | 3.33 | 100.86 |
| HDCA | 5 | 108.36 | 5.20 | 112.76 | 2.60 | 99.34 |
|  | 100 | 100.21 | 3.34 | 100.98 | 3.34 | 96.72 |
|  | 2000 | 99.94 | 2.74 | 101.12 | 3.61 | 92.86 |
| CDCA | 5 | 110.72 | 3.33 | 112.45 | 2.58 | 93.41 |
|  | 100 | 99.83 | 4.28 | 100.35 | 3.20 | 93.72 |
|  | 2000 | 99.09 | 3.02 | 101.37 | 4.85 | 94.31 |
| DCA | 5 | 106.91 | 3.47 | 112.58 | 2.06 | 86.88 |
|  | 100 | 99.88 | 2.22 | 100.30 | 2.91 | 96.51 |
|  | 2000 | 98.11 | 3.15 | 101.29 | 2.70 | 91.85 |
| CA | 5 | 111.37 | 7.47 | 112.27 | 3.12 | 99.70 |
|  | 100 | 100.08 | 2.57 | 100.23 | 3.54 | 99.23 |
|  | 2000 | 98.58 | 2.85 | 100.79 | 3.35 | 92.83 |
| α-MCA | 5 | 113.11 | 6.83 | 114.42 | 4.87 | 97.80 |
|  | 100 | 101.01 | 2.00 | 101.01 | 3.44 | 97.55 |
|  | 2000 | 99.85 | 3.12 | 101.29 | 3.33 | 95.55 |
| β-MCA | 5 | 108.02 | 4.99 | 113.67 | 2.49 | 94.58 |
|  | 100 | 101.12 | 3.27 | 101.12 | 9.82 | 91.96 |
|  | 2000 | 101.23 | 2.19 | 100.37 | 3.87 | 92.46 |
| G-LCA | 5 | 107.73 | 4.37 | 103.08 | 2.71 | 103.33 |
|  | 100 | 100.91 | 2.23 | 100.34 | 3.55 | 104.35 |
|  | 2000 | 103.28 | 2.49 | 104.08 | 3.27 | 106.44 |
| G-UDCA | 5 | 110.83 | 2.14 | 115.55 | 6.38 | 98.55 |
|  | 100 | 100.70 | 1.83 | 100.45 | 4.66 | 96.01 |
|  | 2000 | 105.32 | 3.06 | 104.04 | 3.92 | 99.05 |
| G-CDCA | 5 | 110.62 | 5.69 | 115.39 | 7.14 | 104.73 |
|  | 100 | 101.49 | 8.00 | 100.46 | 5.52 | 104.12 |
|  | 2000 | 102.50 | 2.01 | 104.43 | 3.51 | 99.42 |
| G-DCA | 5 | 115.90 | 3.74 | 117.48 | 7.39 | 100.71 |
|  | 100 | 100.07 | 1.80 | 100.39 | 4.03 | 104.51 |
|  | 2000 | 98.78 | 2.63 | 103.59 | 2.58 | 99.53 |
| G-CA | 5 | 114.95 | 3.56 | 111.71 | 7.78 | 101.17 |
|  | 100 | 100.13 | 2.19 | 100.45 | 4.28 | 98.58 |
|  | 2000 | 99.49 | 2.74 | 103.99 | 3.87 | 81.21 |
| T-LCA | 5 | 110.88 | 4.19 | 113.95 | 9.20 | 96.49 |
|  | 100 | 99.92 | 2.25 | 100.32 | 2.29 | 98.38 |
|  | 2000 | 96.83 | 3.10 | 102.39 | 13.41 | 103.39 |
| T-UDCA | 5 | 109.39 | 2.54 | 115.80 | 8.09 | 99.81 |
|  | 100 | 100.14 | 1.55 | 100.45 | 3.35 | 97.05 |
|  | 2000 | 102.55 | 7.55 | 104.32 | 5.04 | 95.63 |
| T-HDCA | 5 | 113.64 | 4.40 | 114.92 | 8.20 | 99.69 |
|  | 100 | 100.40 | 1.74 | 100.43 | 3.43 | 96.75 |
|  | 2000 | 99.99 | 3.28 | 104.00 | 5.76 | 91.87 |
| T-CDCA | 5 | 115.04 | 2.86 | 115.03 | 8.96 | 103.81 |
|  | 100 | 100.39 | 2.78 | 100.28 | 4.20 | 102.32 |
|  | 2000 | 97.50 | 2.42 | 103.75 | 5.20 | 99.11 |
| T-DCA | 5 | 107.50 | 4.06 | 114.82 | 4.24 | 96.09 |
|  | 100 | 100.65 | 3.91 | 100.44 | 3.82 | 102.29 |
|  | 2000 | 103.99 | 2.11 | 102.69 | 6.16 | 109.95 |
| T-CA | 5 | 114.89 | 3.44 | 110.80 | 8.57 | 101.55 |
|  | 100 | 100.04 | 2.59 | 100.72 | 3.27 | 105.66 |
|  | 2000 | 99.07 | 3.70 | 102.00 | 6.24 | 117.95 |
| T-β-MCA | 5 | 111.67 | 5.97 | 114.40 | 0.02 | 97.91 |
|  | 100 | 100.43 | 1.69 | 100.27 | 2.36 | 95.67 |
|  | 2000 | 102.55 | 1.23 | 105.52 | 3.79 | 89.45 |

**Supplementary Table 3: Primer sequences for qRT-PCR (mice)**

| **Gene** | **Sequence** |
| --- | --- |
| ***Cyp7a1*** | TACTAGATAGCATCATCAAGGAGGCTC  CCATCCTCAAGGTGCAGAGTG |
| ***Cyp7b1*** | GAGCCTATCTACTTCTACAA  TTCTGTGTTCCAATCTGT |
| ***Cyp8b1*** | GATAGGGGAAGAGAGCCACC  TCCTCAGGGTGGTACAGGAG |
| ***Cyp27a1*** | GAAGCCATCACCTATATC  ATAGACTGAGTTCTGGAA |
| ***Fgf15*** | CAGTCTTCCTCCGAGTAGCG  TGAAGACGATTGCCATCAAG |
| ***Fxr*** | GCACGCTGATCAGACAGCTA  CAGGAGGGTCTGTTGGTCTG |
| ***Pxr*** | GTTCAAGGGCGTCATCAACT  TTCTGGAAGCCACCATTAGG |
| ***Car*** | GCTCTTCCGGTCCCTAACC  GACAGAACGTAGTGTTGAGTGAG |
| ***Vdr*** | ACCCTGGTGACTTTGACCG  GGCAATCTCCATTGAAGGGG |
| ***Hnf4α*** | GCTGTCCTCGTAGCTTGACC  TTAAGAAGTGCTTCCGGGCT |
| ***I-Babp*** | CCTTCAGTGGCAAATATG  GACCTCTGTGATGATCTT |
| ***Lrh-1*** | TGAGGAACAACTCCGGGAAAA  CAGACACTTTATCGCCACACA |
| ***Lxr*** | GGCTGCAGGTGGAGTTCATC  AATGAGCAGAGCAAACTCAGCAT |
| ***Shp*** | GTACCTGAAGGGCACGATCC  GTGAAGTCTTGGAGCCCTGGT |
| ***Fgfr4*** | GTACCCTCGGACCGCGGCACATAC  GCCGAAGCTGCTGCCGTTGATG |
| ***β-Klotho*** | CGAGCCCATTGTTACCTTGT  CTCCAAAGGTCTGGAAGCAG |
| ***Ntcp*** | GGTTCTCATTCCTTGCGCCA  GCATCTTCTGTTGCAGCAGC |
| ***Bsep*** | TTCTGTTCTCCACCACTA  GCCATAATGAAGTTGTATGC |
| ***Mrp4*** | GCAAAGCCCATGTACCATCT  ACCACGGCTAACAACTCACC |
| ***Ugt1a1*** | ATGGCTTTCTTCTCCGGAAT  TCAGAAAAAGCCCCTATCCC |
| ***Ugt1a6*** | CACCGGAACTAGACCATCGAA  GCATCATCACCATCGGAACTC |
| ***Ugt1a7*** | TGCAATGGAGTTCCGATGGT  CTGGAGAGGCGCATGATGTT |
| ***Ugt2b34*** | GGAGAATGCCATGCGGTTAT  CTGCCACACGAAGATGCTTG |
| ***Ugt2b35*** | GTGGCGCGAATGGACTCTAT  TCTCAGGTGCTTGGCTCCTT |
| ***Asbt*** | GGAACTGGCTCCAATATCCTG  GTTCCCGAGTCAACCCACAT |
| ***Ostα*** | GTCTCAAGTGATGAACTGCCA  TTGAGTGCTGAGTCCAGGTC |
| ***Ostβ*** | GTATTTTCGTGCAGAAGATGCG  TTTCTGTTTGCCAGGATGCTC |
| ***Gapdh*** | TTGATGGCAACAATCTCCAC  CGTCCCGTAGACAAAATGGT |
